# Quantitative ultrasound radiomics in predicting response to neoadjuvant chemotherapy in patients with locally advanced breast cancer: Results from multi‐institutional study

**DOI:** 10.1002/cam4.3255

**Published:** 2020-06-29

**Authors:** Daniel DiCenzo, Karina Quiaoit, Kashuf Fatima, Divya Bhardwaj, Lakshmanan Sannachi, Mehrdad Gangeh, Ali Sadeghi‐Naini, Archya Dasgupta, Michael C. Kolios, Maureen Trudeau, Sonal Gandhi, Andrea Eisen, Frances Wright, Nicole Look Hong, Arjun Sahgal, Greg Stanisz, Christine Brezden, Robert Dinniwell, William T. Tran, Wei Yang, Belinda Curpen, Gregory J. Czarnota

**Affiliations:** ^1^ Department of Radiation Oncology Sunnybrook Health Sciences Centre Toronto ON Canada; ^2^ Department of Radiation Oncology University of Toronto Toronto ON Canada; ^3^ Physical Sciences Sunnybrook Research Institute Toronto ON Canada; ^4^ Department of Medical Biophysics University of Toronto Toronto ON Canada; ^5^ Department of Electrical Engineering and Computer Sciences Lassonde School of Engineering York University Toronto ON Canada; ^6^ Department of Physics Ryerson University Toronto ON Canada; ^7^ Medical Oncology Department of Medicine Sunnybrook Health Sciences Centre Toronto ON Canada; ^8^ Department of Medicine University of Toronto Toronto ON Canada; ^9^ Surgical Oncology Department of Surgery Sunnybrook Health Sciences Centre Toronto ON Canada; ^10^ Department of Surgery University of Toronto Toronto ON Canada; ^11^ Medical Oncology Saint Michael's Hospital University of Toronto Toronto ON Canada; ^12^ Department of Radiation Oncology Princess Margaret Hospital University Health Network Toronto ON Canada; ^13^ Radiation Oncology London Health Sciences Centre London ON Canada; ^14^ Department of Oncology Schulich School of Medicine and Dentistry Western University London ON Canada; ^15^ Evaluative Clinical Sciences Sunnybrook Research Institute Toronto ON Canada; ^16^ Department of Diagnostic Radiology University of Texas Houston TX USA; ^17^ Department of Medical Imaging Sunnybrook Health Sciences Centre Toronto ON Canada; ^18^ Department of Medical Imaging University of Toronto Toronto ON Canada

**Keywords:** imaging biomarker, locally advanced breast cancer, machine learning, neoadjuvant chemotherapy, quantitative ultrasound, radiomics, response prediction, texture analysis

## Abstract

**Background:**

This study was conducted in order to develop a model for predicting response to neoadjuvant chemotherapy (NAC) in patients with locally advanced breast cancer (LABC) using pretreatment quantitative ultrasound (QUS) radiomics.

**Methods:**

This was a multicenter study involving four sites across North America, and appropriate approval was obtained from the individual ethics committees. Eighty‐two patients with LABC were included for final analysis. Primary tumors were scanned using a clinical ultrasound system before NAC was started. The tumors were contoured, and radiofrequency data were acquired and processed from whole tumor regions of interest. QUS spectral parameters were derived from the normalized power spectrum, and texture analysis was performed based on six QUS features using a gray level co‐occurrence matrix. Patients were divided into responder or nonresponder classes based on their clinical‐pathological response. Classification analysis was performed using machine learning algorithms, which were trained to optimize classification accuracy. Cross‐validation was performed using a leave‐one‐out cross‐validation method.

**Results:**

Based on the clinical outcomes of NAC treatment, there were 48 responders and 34 nonresponders. A *K*‐nearest neighbors (*K‐*NN) approach resulted in the best classifier performance, with a sensitivity of 91%, a specificity of 83%, and an accuracy of 87%.

**Conclusion:**

QUS‐based radiomics can predict response to NAC based on pretreatment features with acceptable accuracy.

## Graphical abstract

This multi‐institutional study investigated the role of radiomics from quantitative ultrasound (QUS) in predicting the final response to neoadjuvant chemotherapy (NAC) for 82 patients with locally advanced breast cancer (LABC). We had shown the QUS‐radiomics model can predict the response to treatment with an accuracy of 87% from spectroscopic features obtained before the start of NAC.

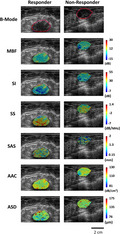

## 1 INTRODUCTION

Locally advanced breast cancer (LABC) includes tumor larger than 5 cm, an extension to the chest wall or skin (regardless of tumor size), and with extensive lymph node involvement.^1^, ^2^ Neoadjuvant chemotherapy (NAC) is the standard of care for patients with LABC, which can downstage tumors leading to breast conservation surgery.^3^ Also, pathological complete response (pCR) is associated with better clinical outcomes compared to nonresponders in certain molecular subgroups. However, only 20%‐40% of patients achieve pCR following NAC^4^ with 40%‐60% having partial responses. Several clinical and molecular features have been identified to be associated with higher rates of response, which include initial tumor size, human epidermal growth factor receptor 2 (HER2) expressing, or triple‐negative tumors.

Image‐based biomarkers have shown success as prognostic and predictive markers for different malignancies and treatment modalities. With the introduction of computer vision in imaging and sophisticated interpretation made possible using machine‐learning classifiers, the application of imaging in oncology has expanded beyond the traditional role of staging and response evaluation.^5^ The establishment of the field of “radiomics” has proven its potential in the noninvasive biological characterization of tumors. The use of ultrasound, magnetic resonance imaging (MRI), computed tomography (CT), and positron emission tomography (PET) for assessing response in patients with breast cancer undergoing NAC have been reported with variable efficacy to date.^6^, ^7^, ^8^

Quantitative ultrasound spectroscopy (QUS) has been used to predict and monitor treatment response in several clinical studies.^8^, ^9^, ^10^ Ultrasound has the benefit of being a relatively inexpensive imaging modality in comparison to MRI and PET and does not emit ionizing radiation or require contrast agents. The conventional use of ultrasound imaging involves “B‐mode” images that are constructed from raw radiofrequency (RF) data. While these images can show some qualitative and quantitative information, much of the frequency‐dependent information is lost with the conversion of RF data. QUS imaging retains this RF data and applies a fast Fourier transform (FFT) to display the data as a frequency spectrum. The analysis of the power spectrum leads to various features like spectral slope (SS), spectral intercept (SI) at 0 MHz, mid‐band fit (MBF), average scatterer diameter (ASD), average acoustic concentration (AAC), attenuation coefficient estimate (ACE), and spacing among scatterers (SAS). In previous studies, it has been demonstrated that QUS features can represent tissue architecture based on scatterer properties reflecting tumor biological behavior as well as ongoing changes associated with treatment.^9^ The QUS values can be obtained from smaller units within the target volume similar to the pixels obtained in cross‐sectional imaging like CT or MRI, which can help in characterizing tumor heterogeneity. Further texture analysis of the QUS parametric maps can unfold valuable information which had been shown to improve the classification performances in predicting treatment response.^11^

In this multi‐institutional study, including four institutions from Canada and the United States, we further explore the effectiveness of QUS‐radiomics in predicting response to NAC for general application.

## 2 METHODS

### 2.1 Patient selection and treatment

The prospective observational study was approved by the individual, institutional ethics committees: Sunnybrook Health Sciences Centre (Toronto, Canada), MD Anderson Cancer Center (Texas, USA), Princess Margaret Cancer Centre (Toronto, Canada), and St. Michael's Hospital (Toronto, Canada). The study had been registered with the clinicaltrials.gov registry (NCT04134780).

All the participants enrolled in the study were required to have histological confirmation of primary breast malignancy without distant metastasis and a decision to undergo NAC by the treating physician. After obtaining appropriate consent, the patients were accrued, treated, and followed up according to standard clinical practice. The study accrual was done from June 2015 to June 2018. NAC consisted primarily of anthracycline and taxane‐based drugs. Following NAC, the decision regarding the type of surgery (breast conservation vs mastectomy) patients underwent was made by the surgeon and oncologists and according to the patients’ wishes. Adjuvant radiotherapy, endocrinal therapy, and targeted therapy were guided by standard institutional protocols.

### 2.2 Response assessment

Participants were classified as either responder (R) or nonresponder (NR) based on results from the surgical specimens by a dedicated breast‐pathologist following mastectomy or lumpectomy using modified RECIST criteria. For this study, participants were identified as responders if they had a pCR or had less than 1% cellularity in the tumor bed (including both invasive and in situ disease). Participants with cellularity greater than 1% but a decrease in size greater than 30% were also classified as responders. Participants with disease progression or a primary tumor size decrease of less than 30% were classified as nonresponders.

### 2.3 Instrumentation and Data Acquisition

Ultrasound imaging was performed before the participant received the first dose of NAC. Participants were scanned with a Sonix RP clinical system (Analogic Medical Corp.) using an L14‐5/60, linear array transducer with a bandwidth range of 3.0‐8.5 MHz and a 6.5 MHz center frequency (n = 63). Nineteen patients were scanned using a GE LOGIC E9 system (GE Healthcare) using an ML6‐15 broad‐spectrum linear matrix array transducer with a bandwidth range of 4.5‐9.9 MHz and a center frequency of 6.9 MHz. B‐mode images were also acquired simultaneously. Further details have been discussed in previous work, and no significant difference was witnessed between the two systems in terms of clinical utility.^8^

### 2.4 QUS data processing

Tumors were contoured to define a region of interest (ROI) from where the QUS parameters were extracted. A sliding window analysis was performed within the ROI involving a 2 mm × 2 mm sliding window with an overlap of 92% in the axial and lateral directions. For each window, an FFT was applied to the RF signal and then normalized to a tissue‐mimicking phantom to produce a normalized power spectrum.

Spectral parameters were determined from the normalized power spectrum using linear regression in a −6 dB frequency bandwidth window.^12^, ^13^ Seven parameters were acquired: SS, SI at 0 MHz, MBF, ASD, AAC, ACE, and SAS. QUS parameters were calculated from each window in the ROI, identified as sub‐ROIs aiding in mapping the spatial heterogeneity. Each feature was separately analyzed to produce individual parametric maps based on the quantitative characterization of the values within the sub‐ROI. Texture analysis was performed on the parametric maps using the gray level co‐occurrence matrix (GLCM) which assessed the relationship between a reference pixel and the neighboring pixels (0°, 90°, 45°, and 135°). Four texture features, contrast (CON), correlation (COR), energy (ENE), and homogeneity (HOM) parameters, were generated for the study.^14^

A total of 24 texture parameters were obtained from six spectral parameters leading to a total of 31 features (texture analysis was not done for ACE). For each patient, the parametric and texture parameters were averaged over 3‐5 tumor slices.

### 2.5 Statistical analysis

Statistical tests were performed to compare if a feature was significantly different between the two groups (R and NR). The Shapiro‐Wilk test was performed to determine the distribution of data. An independent *t* test was performed for normally distributed data, while Mann‐Whitney *U* test was done for others. A *P*‐value of <.05 was considered significant. Patients were classified into the two groups (R vs NR) based on their spectral and texture feature values using machine learning classifiers: K‐nearest neighbors (*K‐*NN*)*, support vector machine with radial basis function kernel (SVM‐RBF), and Fisher's linear discriminant (FLD). For all three machine learning methods, up to 4 of the best classifying features out of 31 were chosen, and classifier parameters were tuned for the best performance. Due to the lower number of responding and nonresponding patients compared to the total number of features, feature reduction was conducted to prevent overfitting and reduce variance. Feature selection was performed using a sequential forward feature selection. The training involved all 82 patients and cross‐validation was performed using the leave‐one‐out method. The predicted and actual responses for each patient were compared to determine the confusion matrix. Receiver operator characteristic curves were generated for each classifier, from which the area under the curve (AUC) was derived. Feature extraction, QUS analysis, and machine learning were performed using MATLAB R2016a (MathWorks). Other statistical tests were performed using IBM SPSS version 22 (IBM Corporation).

## 3 RESULTS

### 3.1 Clinical characteristics and outcomes

There were a total of 82 patients who participated in this study. In total, 50 patients were accrued from Sunnybrook Health Sciences Centre (Toronto, Canada), 7 from Princess Margaret Cancer Centre (Toronto, Canada), 1 from St. Michaels Hospital (Toronto, Canada), and 24 patients from MD Anderson Cancer Centre (Texas, United States of America). The patient's ages ranged from 27 to 74 years (median 52 years). Patient, disease, and related treatment characteristics are summarized in Table 1. Forty‐nine patients were given doxorubicin, cyclophosphamide, and paclitaxel (AC‐T); 30 received fluorouracil, epirubicin, cyclophosphamide, and docetaxel (FEC‐D). Twenty‐seven patients were HER2 positive, 58 were estrogen receptor (ER) positive, and 17 were triple‐negative. All 27 HER2 positive patients received trastuzumab. Forty‐eight patients responded to the NAC, and 34 were classified as nonresponders. A complete pathological response was seen in 17 patients. The characteristics of individual patients are presented in Table S1.

**TABLE 1:** Patient, disease, and treatment characteristics for the patients involved in the study

Features	Frequency
Age	
Mean	50
Median	52 years
Range	27‐74 years
Sex	
Female	80
Male	2
Initial tumor size	
Median:	3.6 cm
Range:	1.2‐11.6 cm
Molecular markers	
ER+	58
PR+	48
HER2+	27
TNBC	17
Histological type	
IDC	66
ILC	7
IMC/Other	8
Chemotherapy	
AC‐T	49
FEC‐D	30
Taxol, no anthracycline	1
Trastuzumab	27
Cisplatin	1
Carboplatin, Taxol	1
Treatment response	
Responder	48
Non‐responder	34

Abbreviations: AC‐T, doxorubicin (Adriamycin) and cyclophosphamide followed by Taxol; ER+/PR+, estrogen/progesterone‐receptor positive status; FEC‐D, 5‐fluorouracil, epirubicin, cyclophosphamide, and docetaxel, trastuzumab: monoclonal antibody (Herceptin); HER2+, human epidermal growth factor receptor 2 positive status; IDC, invasive ductal carcinoma; ILC, invasive lobular carcinoma; IMC, invasive mammary carcinoma; TNBC, triple‐negative breast cancer.

### 3.2 Feature analysis

**FIGURE 1:**
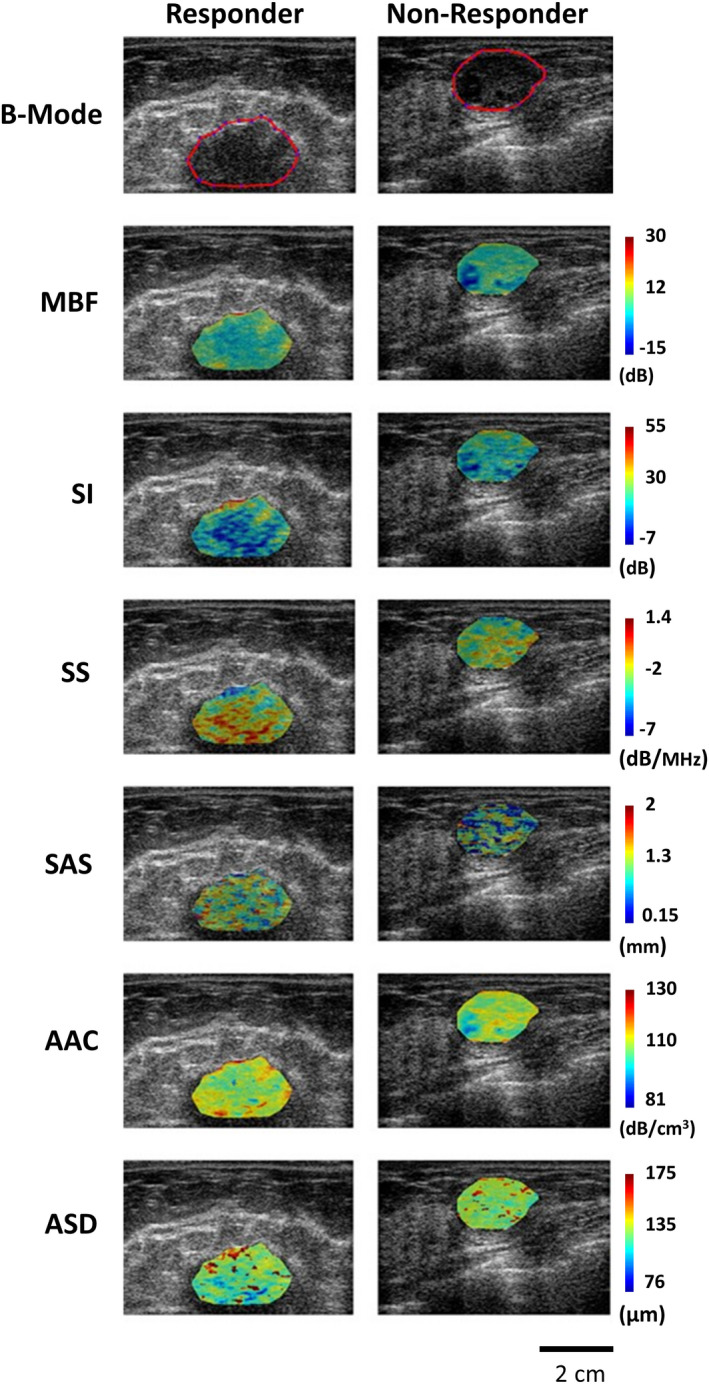
Pretreatment parametric maps of a responder (left panel) and nonresponder (right panel). The top row displays B‐mode images for the responder and nonresponder with the tumor region of interest (ROI) outlined in red. The color images below represent the corresponding spectral parametric maps for two patients (responder vs nonresponder). MBF (dB): mid‐band fit, AAC (dB/cm^3^): average acoustic concentration, ASD (µm): average scatterer diameter, SS (dB/MHz): spectral scope, SAS (mm): spacing among scatterers, ACE (dB/cm‐MHz): attenuation coefficient estimate, SI (dB): 0‐MHz spectral intercept

Pretreatment B‐mode images and parametric maps of MBF, SI, SS, SAS, AAC, and ASD for a representative responding patient and a nonresponding patient are displayed in Figure 1. B‐mode images typically demonstrated hypoechoic appearing tumors. The parametric maps indicate the pixel intensities for each QUS parameter over the entire tumor ROI. Parametric maps appeared as before with obvious differences between responders and nonresponders in addition to obvious heterogeneity within the tumor ROIs.

**FIGURE 2:**
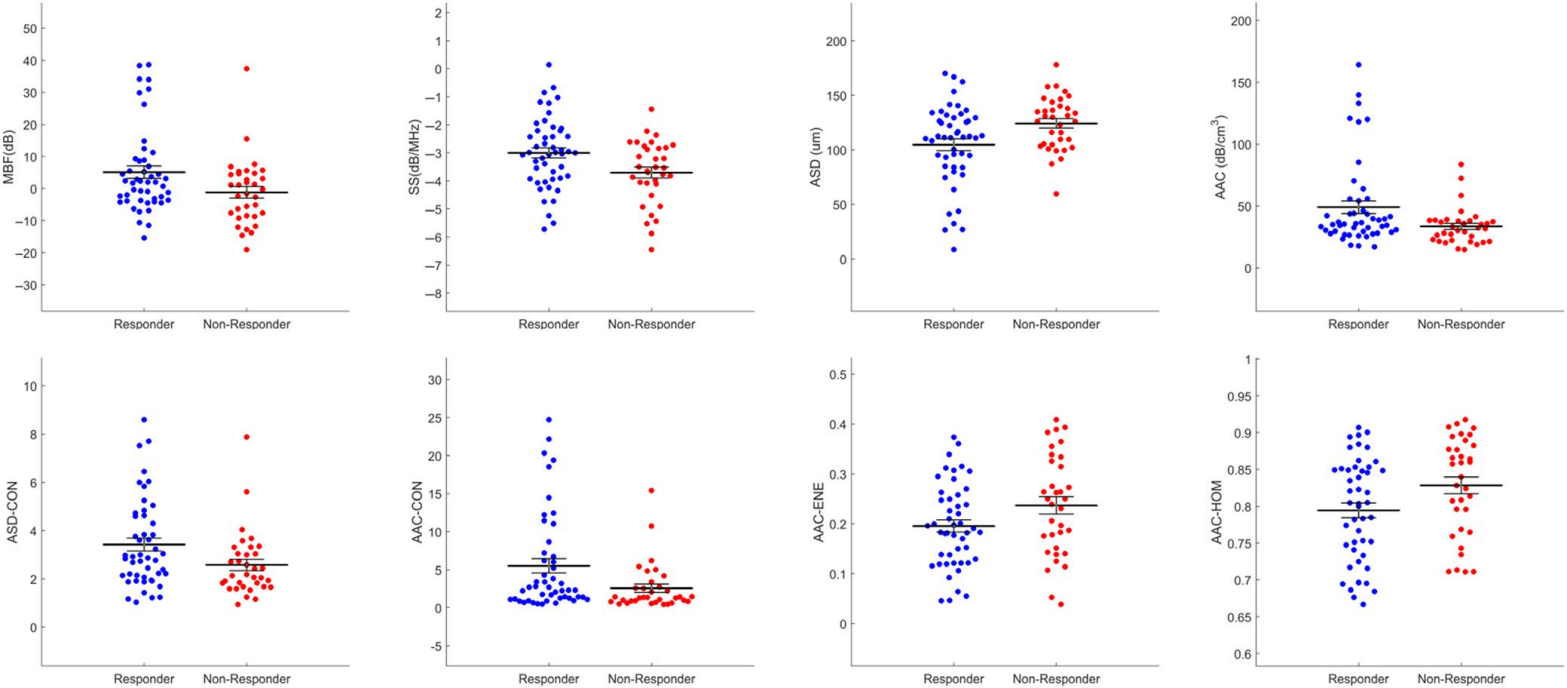
Scatter plots of four spectral and four texture parameter values for responders and nonresponders that were found to have a statistically significant difference from one another (*P* < .05). MBF (dB): mid‐band fit, SS (dB/MHz): spectral slope, ASD (µm): average scatterer diameter, AAC (dB/cm^3^): average acoustic concentration, ASD‐CON: contrast of the average scatterer diameter, AAC‐HOM: homogeneity of the average acoustic concentration, AAC‐ENE: energy of the average acoustic concentration, AAC‐CON: contrast of the average acoustic concentration

Table 2 presents the results of statistical tests performed on the pretreatment QUS spectral and texture data, which compare the difference in QUS values for responding patients and non‐responding patients. The spectral parameters, SS (*P* = .010), MBF (*P* = .043), ASD (*P* = .016), and AAC (*P* = .025) were found to be significantly different between responders and nonresponders. Also, four texture parameters, ASD‐CON (*P* = .018), AAC‐HOM (*P* = .023), AAC‐ENE (*P* = .047), and AAC‐CON (*P* = .015) had a statistically significant difference between responding and nonresponding patients. Figure 2 displays scatter plots of the QUS spectral and texture values that had a statistically significant difference. The majority of the parameters determined were not statistically significant. Scatter plots of all the QUS spectral and texture values are presented in Figure S1. Only tumor core parameters were considered in the study here to evaluate information derived from the bulk tumor mass only.

**TABLE 2:** Mean and SEM of QUS spectral and texture features with a statistically significant difference between responders and non‐responders

Parameter	Mean ± SEM (R)	Mean ± SEM (NR)	*P*‐value
MBF (dB)	5.13 ± 1.92	−1.17 ± 1.78	.043
SS (dB/MHz)	−3.00 ± 0.18	−3.70 ± 0.20	.010
ASD (µm)	104.80 ± 5.36	124.40 ± 4.20	.016
AAC (dB/cm^3^)	49.10 ± 5.04	33.54 ± 2.52	.025
ASD‐CON (AU)	3.43 ± 0.26	2.58 ± 0.23	.018
AAC‐HOM (AU)	0.795 ± 0.010	0.829 ± 0.011	.023
AAC‐ENE (AU)	0.20 ± 0.01	0.24 ± 0.02	.047
AAC‐CON (AU)	5.52 ± 0.93	2.58 ± 0.54	.015

Abbreviations: AAC (dB/cm^3^), average acoustic concentration; ASD (µm), average scatterer diameter; CON, contrast; ENE, energy; HOM, homogeneity; MBF (dB) , mid‐band fit; SEM, standard error of the mean; SS (dB/MHz), spectral slope.

### 3.3 Classifier results

**FIGURE 3:**
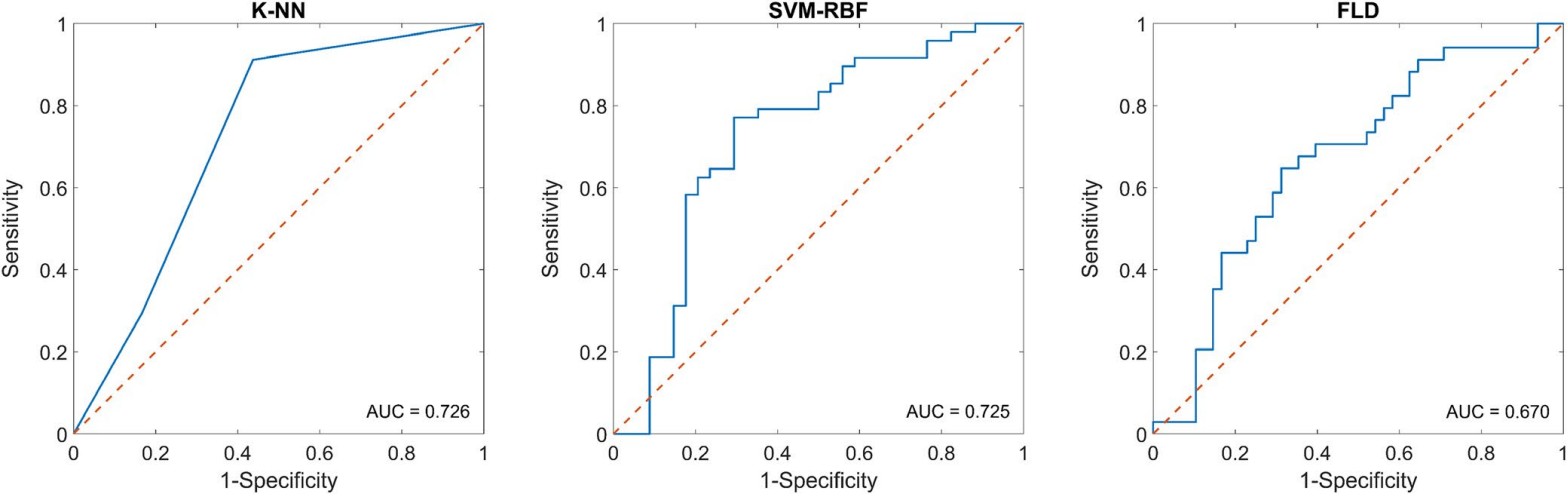
Receiver operating characteristic curve of pretreatment prediction using three classifiers. FLD: Fisher's linear discriminant, *K‐*NN: *K‐*nearest neighbors, SVM‐RBF: support vector machine with radial basis function kernel

The performance results of the classification algorithms made are presented in Table 3. For classification purposes, a *K*‐NN approach was compared to SVM‐RBF and FLD methodology. The corresponding receiver operating characteristic curves comparing the three algorithms evaluated are displayed in Figure 3. The *K*‐NN algorithm had the best performance using three features (AAC‐HOM, SI‐ENE, and SAS‐ENE). It produced the greatest sensitivity (91%), specificity (83%), accuracy (87%), AUC (0.73), and F1‐Score (0.87). The SVM‐RBF algorithm had a lower sensitivity (71%), specificity (80%), accuracy (76%), AUC (0.72), and F1‐score (0.75) compared to *K*‐NN. The FLD algorithm had the lowest values of sensitivity (68%), specificity (65%), accuracy (66%), AUC (0.67), and F1‐score (0.66).

**TABLE 3:** Machine learning classifier performances for the different algorithms

Classifier	%S_n_	%S_p_	AUC	%Acc	F1‐score	Features
*K*‐NN	91.2	83.3	0.726	86.6	0.871	AAC‐HOM, SI‐ENE, SAS‐ENE
SVM‐RBF	70.6	79.2	0.725	75.6	0.746	AAC, SS, SAS‐HOM, SAS‐COR
FLD	67.7	64.6	0.670	65.9	0.661	ASD, SAS‐COR, SAS

Abbreviations: AAC (dB/cm^3^), average acoustic concentration; Acc, accuracy; ASD (µm), average scatterer diameter; AUC, area under curve; COR, correlation; ENE, energy; FLD, Fisher's linear discriminant; HOM, homogeneity; *K‐*NN, *K‐*nearest neighbors; SAS (mm), spacing among scatterers; SI (dB), spectral intercept; S_n_, sensitivity; S_p_, specificity; SS (dB/MHz), spectral slope; SVM‐RBF, support vector machine with radial basis function kernel.

## 4 DISCUSSION

The groundwork for using QUS to predict and monitor treatment was established by a preclinical study showing that cells undergoing apoptosis and exhibiting effects such as nuclear condensation and fragmentation had a 25‐fold increase in ultrasound backscatter intensity compared to normal cells.^15^ Further studies followed, incorporating a greater number of acoustic features used for detecting cell death and allowing for prediction of response to chemotherapy and radiation therapy in vivo.^16^, ^17^, ^18^ The use of this methodology has included differentiating benign from malignant tissue, characterizing tumor grade, and predicting tumor response to treatment in LABC and head and neck cancer patients.^19^

The work here demonstrates that pretreatment QUS‐radiomics can be used as a biomarker for predicting response to NAC. The participation of patients from different institutions increases the robustness of the model and reliability. The *K‐*NN classifier was found to be the best performing classifier for the pretreatment QUS data. The SVM‐RBF was slightly less accurate than *K‐*NN methodology, which may indicate that the data does not exhibit a distribution that can be well separated using a hyperplane. The FLD classifier had the lowest accuracy, which could be due to its reliance on a linear distribution for data that cannot be separated using a linear hyperplane.

The results here, in this multi‐institutional study, were found to be equivalent to single‐institution work reported previously.^8^ The work here used a smaller multi‐institutional set of data collected within a set period of time. The analysis focused on tumor core alone, not incorporating rim features to focus on the performance of the tumor‐alone analysis. The work did not use a balancing approach used in larger data sets given the limited number of patients in the multi‐institutional data here.

Using *K‐*NN methodology, the best features to classify responders and nonresponders were identified as the AAC‐HOM, SI‐ENE, and SAS‐ENE parameters. The AAC is related to the number density of scatterers and the relative acoustic impedance of scatterers in the medium. The SI is related to the composition and distribution of scatterers, and the SAS is related to the distance between regularly spaced scatterers. This suggests that the spatial organization and composition of tumor constituents are important response predictors. Tumor environments are known to be heterogeneous and chaotic compared to normal tissue.^20^, ^21^ A tumor is comprised of a variety of cell types, including fibroblasts, immune cells, adipocytes, and epithelial cells. Due to cell cycles and cell proliferation, these cells will have variable sizes across the tumor. There may also be variations in interstitial fluid pressure due to the tumor's disorganized and leaky vasculature.^22^ These tumor properties may make it difficult to adequately deliver chemotherapeutic drugs throughout the tumor and could result in poor response.^23^, ^24^ Similarly, the expression of molecular features, such as ER, progesterone receptor (PR), and HER2, are linked to different response rates and different overall survival rates for patients receiving chemotherapy.^25^, ^26^, ^27^ Response prediction using image‐based biomarkers has been previously studied using other imaging modalities. Thibault *et al* used DCE‐MRI to determine pharmacokinetic parameters and semiquantitative metrics from breast tumors in patients receiving NAC. Texture features were derived from these parameters, and class analysis was used to predict responders and nonresponders before treatment and within one cycle of chemotherapy.^6^ In another study by Lundgren *et al*, who used texture features derived from DCE‐MRI parameters to predict patient response to NAC after four cycles of chemotherapy.^28^ Diffusion‐weighted MRI (DW‐MRI) has been used to predict pCR in breast cancer patients receiving NAC by detecting changes in intra‐tumoral cellularity.^29^ ^18^F‐FDG‐PET/MRI has also been used recently for breast cancer patients receiving NAC for response prediction.^7^ Whereas MRI‐ and PET‐derived biomarkers have resulted in good results at predicting patient response to NAC early into treatment, compared to QUS, those methodologies are more expensive, have longer image acquisition time, are less portable, and may require the use of contrast agents.^30^

Currently, several months are typically required to determine if a patient is responding to treatment. The pathological response is the gold standard for evaluating the ultimate response to treatment and can only be assessed after chemotherapy and surgery have been completed. QUS methodology has been demonstrated to have the ability to predict response and potentially can be used to assist patients and oncologists in personalizing a course of treatment. Patients who are predicted to be nonresponders could have a modified chemotherapy regime, or proceed directly to surgery, or investigate other treatment options. Early knowledge of patient response to chemotherapy allows for early intervention and potential adaptation for a more personalized therapy.^31^

While the prediction accuracy of our algorithm using *K*‐NN is high using an internal cross‐validation method, it will likely be improved through the incorporation of pretreatment QUS data from a higher number of patients for a more robust prediction algorithm. In addition, with increased patient numbers, potentially individual models for each luminal type can be created to explore if they can lead to further improvements in the classifier performances.

## 5 CONCLUSION

Pretreatment QUS data from multiple healthcare institutions can be used to predict patient response to NAC with an accuracy of 87%. The ability to predict response to NAC with high accuracy before treatment initiation can be adopted by the clinicians for risk stratification and guiding treatment and will lead its way to precision oncology in the future.

## Conflicts of interest

None of the authors have conflicts of interest to declare.

## Author contributions

DD, KQ, KF, DB, LS, MG, AS, AD, MCK, WTT: Investigation, resources, formal analysis, methodology, writing—original draft, review, editing; MT, SG, AE, FW, NLH, AS, GS, CB, RD, WY, BC: Resources, formal analysis, methodology, writing—original draft, review, editing; GJC: conceptualization, investigation, resources, project administration, formal analysis, methodology, writing—original draft, review, editing.

## Supporting information

Figure S1

Table S1

## Data Availability

Anonymized data will be made available in accordance with institutional policies.
