# Supplementary material for: Quantitative ultrasound radiomics in predicting response to neoadjuvant chemotherapy in patients with locally advanced breast cancer: Results from multi‐institutional study
Source: Cancer Med. 2020 Jun 29;9(16):5798–806. doi: 10.1002/cam4.3255 (PMC7433820; doi:10.1002/cam4.3255)
Supplement: Supplementary file 1 — Figure S1 [file CAM4-9-5798-s001.docx]

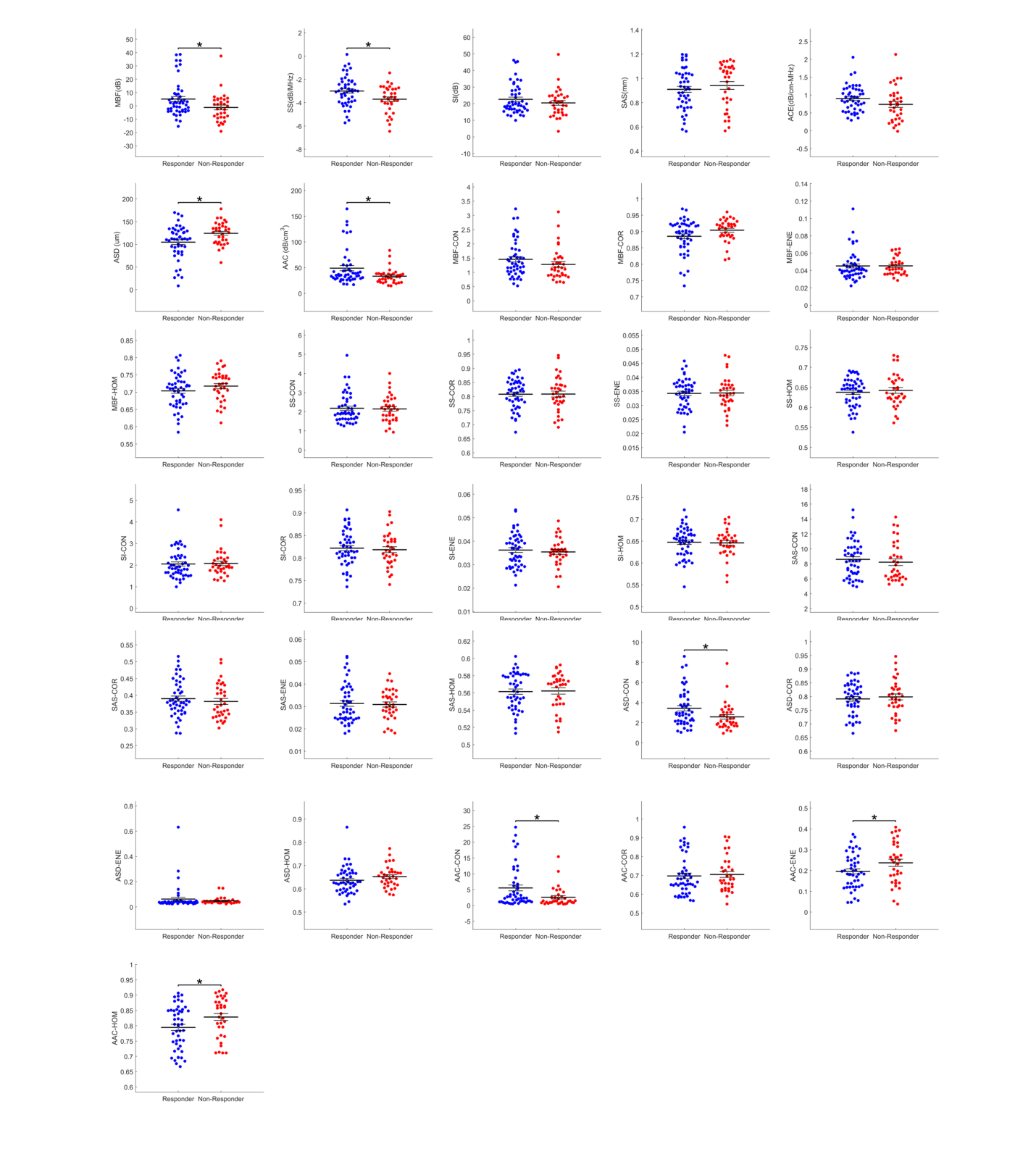


Supplementary Figure 1. Scatter plots of spectral and texture parameter values for responders and non‑responders. * indicates parameters that have a statistically significant difference (p < 0.05). MBF (dB): mid-band fit, SS (dB/MHz): spectral scope, SI (dBr): spectral intercept, AAC (dB/cm^3^): average acoustic concentration, ASD (µm): average scatterer diameter, SAS (mm): spacing among scatterer, ACE (dB/cm-MHz): attenuation coefficient estimate
