# Supplementary material for: Quantitative ultrasound radiomics in predicting response to neoadjuvant chemotherapy in patients with locally advanced breast cancer: Results from multi‐institutional study
Source: Cancer Med. 2020 Jun 29;9(16):5798–806. doi: 10.1002/cam4.3255 (PMC7433820; doi:10.1002/cam4.3255)
Supplement: Supplementary file 2 — Table S1 [file CAM4-9-5798-s002.docx]

Supplementary Table 1: Characteristic information for all patients enrolled in the study.

| **Patient-ID** | **Age** | **Sex** | **ER** | **PR** | **HER2** | **Pre-Tx Largest Dimension (cm)^a^** | **Chemo Regimen** | **Trustuzumab** | **Post-Tx largest dimension (cm)^b^** | **mRECIST** | **Response** | |
| --- | --- | --- | --- | --- | --- | --- | --- | --- | --- | --- | --- | --- |
| MD Anderson | | | | | | | | | | | | |
| 1 | 56 | F | + | - | + | 5.2 | AC-T | Yes | 0 | 5 | R |  |
| 2 | 61 | F | + | + | - | 3.8 | AC-T | No | 5.3 | 1 | NR |  |
| 4 | 58 | F | + | + | - | 3.2 | AC-T | No | 3.5 | 1 | NR |  |
| 6 | 51 | F | + | + | + | 4.1 | AC-T | Yes | 1.6 | 3 | R |  |
| 7 | 39 | F | + | + | - | 8.7 | AC-T | No | 12 | 1 | NR |  |
| 8 | 59 | F | - | - | + | 3.1 | T | Yes | 0 | 5 | R |  |
| 9 | 35 | F | + | + | - | 10 | AC-T | No | 0 | 5 | R |  |
| 10 | 74 | F | - | - | - | 4.5 | AC-T | No | 1.4 | 3 | R |  |
| 11 | 58 | F | - | - | - | 3 | AC-T | No | 3.4 | 1 | NR |  |
| 12 | 41 | F | + | - | - | 1.8 | AC-T | No | 0 | 5 | R |  |
| 13 | 57 | F | - | - | - | 7.6 | AC-T | No | NA | 5 | NR |  |
| 14 | 46 | F | + | - | - | 6 | AC-T | No | 3.5 | 3 | R |  |
| 15 | 56 | F | + | + | - | 4.1 | AC-T | No | 3 | 2 | NR |  |
| 16 | 45 | F | - | + | - | 3.4 | AC-T | No | 3.5 | 1 | NR |  |
| 17 | 52 | F | + | + | - | 3.6 | AC-T | No | NA | 1 | NR |  |
| 18 | 37 | F | + | + | - | 3.9 | AC-T | No | 1.3 | 3 | R |  |
| 19 | 38 | F | + | + | - | 3 | AC-T | No | 4 | 1 | NR |  |
| 20 | 52 | F | + | + | - | 3.4 | AC-T | No | 1.5 | 3 | R |  |
| 21 | 66 | F | + | + | - | 2.9 | AC-T | No | 1 | 3 | R |  |
| 22 | 57 | F | + | + | - | 3.1 | AC-T | No | 1.9 | 3 | R |  |
| 24 | 44 | F | + | + | - | 1.8 | AC-T | No | 1.8 | 1 | NR |  |
| 25 | 35 | F | + | - | - | 2.4 | AC-T | No | 1 | 3 | R |  |
| 26 | 49 | F | + | - | - | 6.1 | AC-T | No | 0 | 5 | R |  |
| 27 | 28 | F | - | - | - | 2.7 | CDDP | No | 2 | 2 | NR |  |
| Princess Margaret | | | | | | | | | | | | |
| 1 | 58 | F | + | + | - | 4.4 | FEC-D | No | 3 | 3 | R |  |
| 2 | 62 | F | - | - | - | 2.9 | AC-T | No | 1.5 | 3 | R |  |
| 3 | 40 | F | - | - | - | 4 | FEC-D | No | 0 | 5 | R |  |
| 4 | 54 | F | + | + | - | 2.5 | FEC-D | No | 1 | 3 | R |  |
| 5 | 36 | F | - | + | - | 4.4 | AC-T | No | 0.3 | 4 | R |  |
| 6 | 65 | F | + | - | + | 2.1 | FEC-D | Yes | 2 | 2 | R | ^c^ |
| 10 | 38 | F | + | + | + | 1.9 | FEC-D | Yes | 0.45 | 3 | R |  |
| St. Michael's Hospital | | | | | | | | | | | | |
| 1 | 33 | F | + | + | - | 2.9 | FEC-D | - | NA^d^ | - | NR |  |
| Sunnybrook Health Sciences Centre | | | | | | | | | | | | |
| 133 | 55 | F | + | + | - | 8.4 | FEC-D | No | 8 | 2 | NR |  |
| 134 | 45 | F | + | + | + | 5.9 | FEC-D | Yes | 5.5 | 2 | NR |  |
| 135 | 53 | F | - | - | - | 3.1 | AC-T | No | 2.7 | 2 | NR |  |
| 136 | 39 | F | + | - | + | 7 | FEC-D | Yes | 5.5 | 2 | NR |  |
| 137 | 57 | F | - | - | + | 4.8 | FEC-D | Yes | 5.5 | 1 | R | ^c^ |
| 138 | 50 | F | + | + | + | 4.1 | FEC-D | Yes | 1.9 | 3 | R |  |
| 139 | 62 | F | - | - | - | 2.1 | AC-T | No | 3 | 1 | R | ^c^ |
| 140 | 69 | F | + | + | + | 7.3 | AC-T | Yes | 4.5 | 3 | R |  |
| 141 | 69 | M | + | + | - | 3.1 | FEC-D | No | 2.2 | 2 | NR |  |
| 142 | 36 | F | + | - | + | 6.3 | FEC-D | Yes | 10 | 1 | R | ^c^ |
| 143 | 42 | F | + | + | + | 6 | FEC-D | Yes | 7 | 1 | NR |  |
| 144 | 72 | M | + | + | + | 1.2 | AC-T | Yes | 2 | 1 | R | ^c^ |
| 145 | 70 | F | + | + | - | 3.7 | FEC-D | No | 14 | 1 | NR |  |
| 148 | 45 | F | + | + | - | 2 | FEC-D | No | 1.5 | 2 | NR |  |
| 149 | 42 | F | + | + | - | 3.8 | AC-T | No | 3.7 | 2 | R | ^c^ |
| 150 | 63 | F | + | + | - | 8.6 | AC-T | No | 7.5 | 2 | NR |  |
| 151 | 52 | F | - | - | - | 3.1 | FEC-D | No | 0.2 | 4 | R |  |
| 152 | 61 | F | + | + | - | 8.2 | AC-T | No | 10 | 1 | NR |  |
| 153 | 54 | F | - | - | - | 2.3 | FEC-D | No | 1.5 | 3 | R |  |
| 154 | 68 | F | + | + | + | 2.2 | AC-T | Yes | 2.5 | 1 | R | ^c^ |
| 156 | 46 | F | + | + | + | 2.6 | FEC-D | Yes | 2 | 2 | NR |  |
| 157 | 60 | F | - | - | + | 4 | AC-T | Yes | 6 | 1 | NR |  |
| 158 | 57 | F | - | - | + | 3.3 | FEC-D | Yes | 2.5 | 2 | R | ^c^ |
| 159 | 54 | F | + | - | + | 2.1 | AC-T | Yes | 2 | 2 | R | ^c^ |
| 160 | 67 | F | + | + | + | 2.5 | AC-T | Yes | 1.4 | 3 | R |  |
| 161 | 55 | F | - | - | - | 2.7 | AC-T | No | 2.5 | 2 | R | ^c^ |
| 162 | 51 | F | + | + | + | 2.7 | FEC-D | Yes | 2.5 | 2 | NR |  |
| 163 | 45 | F | + | + | - | 2.4 | FEC-D | No | 1.9 | 2 | NR |  |
| 164 | 45 | F | - | - | - | 3.3 | AC-T | No | 3.3 | 1 | NR |  |
| 165 | 56 | F | + | + | - | 2.9 | FEC-D | No | 2.2 | 2 | NR |  |
| 166 | 42 | F | + | + | + | 2.1 | FEC-D | Yes | 1 | 3 | R |  |
| 168 | 38 | F | - | - | + | 2.2 | AC-T | Yes | 2 | 2 | R | ^c^ |
| 169 | 36 | F | + | + | + | 1.9 | FEC-D | Yes | 1.2 | 3 | R |  |
| 170 | 27 | F | + | + | + | 11.3 | AC-T | Yes | 4.5 | 3 | R |  |
| 171 | 36 | F | + | + | - | 9.8 | FEC-D | No | 15 | 1 | NR |  |
| 172 | 69 | F | + | + | - | 8.8 | FEC-D | No | 15.7 | 1 | NR |  |
| 173 | 37 | F | + | + | + | 9.6 | AC-T | Yes | 0.8 | 4 | R |  |
| 174 | 50 | F | + | + | + | 5.7 | AC-T | Yes | 3 | 3 | R |  |
| 175 | 36 | F | - | - | - | 5.1 | AC-T | No | 2.5 | 3 | R |  |
| 179 | 62 | F | + | + | - | 3.5 | FEC-D | No | 2.6 | 2 | NR |  |
| 180 | 63 | F | - | - | - | 3.7 | AC-T | No | 3 | 2 | NR |  |
| 181 | 53 | F | + | + | - | 3.7 | AC-T | No | NA^d^ | 3 | R | ^c^ |
| 183 | 55 | F | + | - | - | 3.4 | AC-T | No | 1.5 | 3 | R |  |
| 184 | 47 | F | - | - | - | 3.9 | AC-T | No | 2.5 | 3 | R |  |
| 185 | 31 | F | + | + | - | 5.3 | AC-T | No | 0.45 | 4 | R |  |
| 186 | 34 | F | + | + | - | 4.1 | FEC-D | No | 0.35 | 4 | R |  |
| 187 | 71 | F | - | - | - | 3.6 | AC-T | No | 3 | 2 | NR |  |
| 188 | 46 | F | + | - | + | 11.6 | FEC-D | Yes | 9.8 | 2 | R | ^c^ |
| 189 | 47 | F | + | - | - | 5.5 | AC-T | No | 2.7 | 3 | NR | ^e^ |
| 190 | 34 | F | - | - | - | 1.9 | CT | No | 1.3 | 3 | R |  |

**Abbreviations:** ER+/PR+: estrogen/progesterone-receptor positive status, HER2+: human epidermal growth factor receptor 2 positive status, AC-T: doxorubicin (Adriamycin) and cyclophosphamide followed by Paclitaxel, FEC-D: 5-fluorouracil, epirubicin, cyclophosphamide, and docetaxel, trastuzumab: monoclonal antibody (Herceptin), T: Paclitaxel, no anthracycline, CDDP: Cisplatin CT: Platinum and Taxel.

^a^ Maximum tumour size measured clinically and radiologically before initiation of any treatment.

^b^ Maximum dimension of the viable focus of tumour or span of the tumour bed (when multiple foci of residual tumour) from the pathological specimen.

^c^ Tumor size decreased less than 30% but post treatment cellularity is very low. The patients were considered to be a responder to NAC

^d^ “NA“indicates not assessable tumour size of final response. In such cases the labels (R vs. NR) was decided by the treating oncologist/tumour board.

^e^ Tumor size decrease greater than 30% but post treatment cellularity is very high. The patient was considered to be a non-responder to NAC
